# Clinical significance of securin expression in solid cancers: A PRISMA-compliant meta-analysis of published studies and bioinformatics analysis based on TCGA dataset

**DOI:** 10.1097/MD.0000000000030440

**Published:** 2022-09-16

**Authors:** Xiang Liu, Wei Zeng, Dayang Zheng, Min Tang, Wangyan Zhou

**Affiliations:** a Department of Cardiothoracic Surgery, the Second Affiliated Hospital, University of South China, Hengyang, China; b Department of Medical Humanities and Education Department, the First Affiliated Hospital, University of South China, Hengyang, China.

**Keywords:** cancer, metastasis, prognosis, securin

## Abstract

Numerous studies have investigated the clinical significance of securin expression in solid cancers; however, the results have been inconsistent. Hence, we performed a meta-analysis of published studies to assess the clinical value of securin expression in patients with solid cancers.

**Methods:**

The Chinese National Knowledge Infrastructure, Web of Science, PubMed, and EMDASE databases were searched for eligible studies (from inception up to April 2021). Bioinformatics analysis based on The Cancer Genome Atlas dataset was also performed to evaluate the prognostic value of securin expression.

**Results:**

A total of 25 articles with 26 studies were included in the meta-analysis. The results of the meta-analysis implied that high securin expression was positively correlated with unfavorable overall survival (OS) (hazard ratio = 1.52, 95% CI, 1.33–1.73; *P* < .001) and lymph node metastasis (odd ratio = 2.96, 95% CI, 2.26–3.86; *P* < .001). Consistently, our bioinformatics analysis showed that increased securin expression was associated with worse OS and shorter disease-free survival in cancer patients.

**Conclusion:**

Our study indicated that securin overexpression was positively associated with metastasis and inversely related to the prognosis of patients with solid cancers. However, additional high-quality studies should be conducted to validate these findings.

**Key Points**

- High securin expression is closely associated with poor overall survival in solid cancers.
- There is a negative correlation between securin expression and disease-free survival in solid cancers.
- High securin expression is positively related to tumor lymph node metastasis.

## 1. Introduction

Securin is a multifunctional protein with 202 amino acids encoded by a gene located on chromosome 5 (5q35.1), which is also called pituitary tumor transforming gene-1 and comprises 4 introns and 5 exons.^[1,2]^ Securin is normally expressed in tissues with high proliferative activity, including the spleen, thymus gland, and testis, whereas it is rarely expressed in differentiated mature tissues.^[3]^ Securin is a multifunctional protein that is involved in a broad spectrum of cellular physiological activities. First, securin has been revealed to negatively mediate sister chromatid separation, thereby maintaining the homeostasis of cellular mitosis. Mechanistically, the securin protein in its complete form can inhibit separase via direct binding of its C-terminal region over the surface of separase, which, once activated, results in cleavage of the cohesin rings that sustain chromosomes together.^[4]^ Additionally, securin can form a complex with GM130, AKAP450, and γ-tubulin to facilitate centrosomal and noncentrosomal microtubule nucleation in different cell types, participating in the regulation of cell migration.^[5]^ Conversely, because of its inhibitory effect on chromosome segregation, which has been proposed as a key source of genetic instability, and promotive effect on cell migration, securin is somewhat anticipated to exhibit tumorigenic activity.^[6]^ In recent years, mounting evidence has demonstrated that securin is aberrantly upregulated in various human cancers and exerts critical oncogenic functions.^[7,8]^ For example, in lung cancer, the downregulation of securin inhibits tumor cell growth by inactivating the TGFβ1/SMAD3 signaling pathway.^[9]^ In cholangiocarcinoma, securin overexpression can facilitate tumor cell proliferation via activation of mitogen activated protein kinase (MAPK).^[10]^ In esophageal squamous cell cancer, securin upregulation contributes to cell motility and metastasis by increasing the expression of several members of the Ras and Rho families^[2]^ and via c-myc-mediated galectin-1 transactivation.^[11]^ Furthermore, securin was observed to induce epithelial-mesenchymal transition (EMT) in esophageal squamous cell cancer cells by activating the expression of glioma-associated oncogene homolog1.^[12]^ In hepatocellular carcinoma (HCC), securin can negatively regulate cellular apoptosis via inactivation of p53 and p38, as well as by activating the protein kinase B (AKT) signaling pathway.^[13,14]^ Moreover, securin was reported to promote HCC cell proliferation by upregulating c-Myc.^[15]^ In colorectal cancer, securin can lead to genetic instability by inhibiting Ku70 activity and disturbing the nonhomologous end-joining dsDNA repair pathway.^[16]^ Overall, the literature supports the notion that securin functions as an oncogene in human cancers.

In view of securin-mediated oncogenic functions, a large number of studies have been conducted to explore whether securin can be used as a prognostic biomarker and therapeutic target. Nevertheless, these studies have yielded conflicting results regarding the prognostic significance of securin in human cancers. Most studies indicate that high securin expression is closely correlated with poorer overall survival (OS) and even acts as an independent prognostic biomarker in patients with malignant tumors, such as esophageal cancer,^[2]^ HCC,^[17,18]^ endometrial cancer,^[19,20]^ glioma,^[21]^ gastric cancer,^[22]^ laryngeal cancer,^[23,24]^ lung cancer,^[9,25]^ colorectal cancer,^[26]^ renal cell cancer,^[27]^ osteosarcoma, and bladder cancer.^[28]^ Conversely, one individual study has shown that the securin expression level is not related to survival outcome in cancer patients.^[28]^ In fact, most studies on securin in cancers were performed in a single center and were limited by a small sample size, which may have yielded biased results. Therefore, we performed a meta-analysis of the current literature to systematically assess whether securin can be used as a prognostic biomarker in pan-cancer. Tumor metastasis is a key event that directly affects survival outcomes in cancer patients^[29,30]^; therefore, the association of securin with metastasis has attracted much attention as well. To date, a convincing conclusion regarding the relationship between securin expression and metastasis has not yet been made. Thus, in this meta-analysis, we conducted a combined analysis to evaluate the association between securin expression and lymph node metastasis (LNM).

## 2. Materials and Methods

This meta-analysis was conducted in accordance with the guidelines of the Preferred Reporting Items for Systematic Reviews and Meta-Analyses.^[31]^ This study is a meta-analysis and bioinformatics analysis; therefore, there is no need to obtain ethical approval.

### 2.1. Search strategy

Four electronic databases, including the Chinese National Knowledge Infrastructure, Web of Science, PubMed, and EMDASE, were searched for eligible studies from inception until April 2021. The key words used for study research were as following: (“pituitary tumor transforming 1” or “PTTG1” or “securin”), and (“neoplasm” or “tumor” or “malignancy” or “cancer”), and (“survival” or “prognostic” or “prognosis” or “outcome”). Besides, manual searches were also performed by screening the reference lists of the relevant publication to identify additional studies. Articles published in full text and in English or Chinese were included.

### 2.2. Inclusion and exclusion criteria

Studies were selected based on the following criteria: solid cancers were diagnosed by histopathology; studies assessed the association between securin expression and OS or LNM; securin expression in cancerous tissues was detected using immunohistochemistry (IHC) staining or polymerase chain reaction (PCR). Moreover, patients were allocated into “low” and “high” groups based on securin expression level; hazard ratios (HRs) or odd ratios (ORs) with their 95% confidence intervals (95% CIs) can be extracted directly or estimated with available information. Study exclusion criteria were as followings: HRs and corresponding CIs could not be obtained owing to insufficient information; populations were not allocated into 2 “low” and “high” groups based on securin expression; the relationships of securin expression to OS or LNM were analyzed using data derived from The Cancer Genome Atlas (TCGA) and Gene Expression Omnibus; Publications were editorials, abstracts, comments, reviews, or animal experiments; or sample size of studies was <30.

### 2.3. Data extraction and quality assessment

Data from each eligible study were independently assessed and extracted by 2 authors (XL and WZ). The collected data items were as following: cancer type, first author’s family name, publication year, country, sample size, age, gender, tumor stage, LNM, OS, detection method, definition of high securin expression, HR with 95% CI and analysis method. Of note, if studies provided HR estimations from COX univariate and multivariate analysis simultaneously, the latter were used for meta-analysis. Two independent investigators (XL and WZ) evaluated the quality of the included studies using the Newcastle Ottawa Scale.^[32]^

### 2.4. Bioinformatics analysis based on TCGA data

We performed Gene Expression Profiling Interactive Analysis (GEPIA) based on TCGA dataset to further assess the prognostic value of securin expression in patients with solid cancers.^[33]^ In this analysis, the Kaplan–Meier method and log-rank test were used to compare survival outcomes between patients with high securin expression and those with low expression, and HRs with 95% CIs and *P* values were presented using the Kaplan–Meier method curve, as previously described.^[34]^

### 2.5. Statistical analysis

All statistical analyses were conducted using Stata 12.0 (STATA Corp., College Station, TX) software. The combined HR and its 95% CI was calculated to assess the association between securin expression and OS. The combined HR and 95% CI >1 implied unfavorable survival outcome patients with high securin expression. The synthesized OR and its 95% CI were calculated to evaluate the relationship between securin expression and LNM. Heterogeneity among eligible studies was analyzed by chi-squared *Q* test and *I*-squared (*I*^2^) statistical test. When *I*^2^ was > 50% or *P* was < .05, the heterogeneity was proposed to be significant, and the random effects model was adopted for conducting the combined analysis; otherwise, the fixed effects model was used. Subgroup and meta-regression analyses were conducted based on similar variables among eligible studies. The stability of the combined results was assessed using a sensitivity analysis conducted by sequentially deleting each individual study. Publication bias was visually evaluated with a funnel plot and statistically assessed using Egger’s test.^[35,36]^ Once significant publication bias was detected, the trim-and-fill method was used to confirm the robustness of the combined results.^[37]^

## 3. Results

### 3.1. Literature search and selection

**Figure 1.**
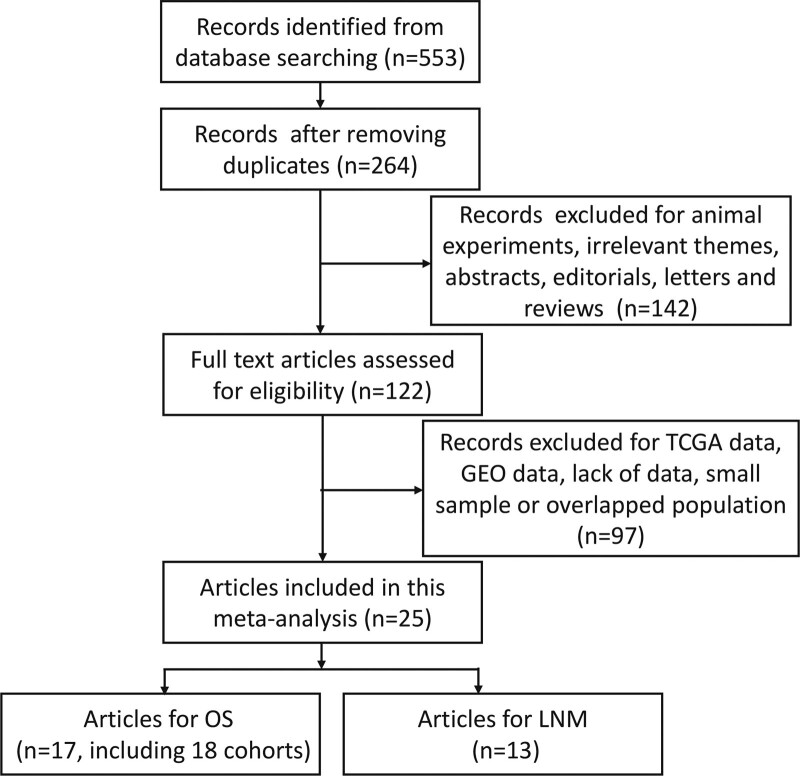
Flowchart of the retrieval and selection of eligible studies. GEO = Gene Expression Omnibus, LNM = lymph node metastasis, OS = overall survival, TCGA = The Cancer Genome Atlas.

To begin with, we obtained a total of 553 publications through retrieving electronic databases. Then, we removed 289 duplicated records with the help of EndNote software. Next, we carefully reviewed the remaining publications by title and abstract, consequently excluding 142 records due to animal studies, irrelevant themes, conference abstracts, editorials, letters, and reviews. Furthermore, we screened the remaining publications by full text and excluded additional 97 publications for TCGA or CEO data, lack of data, small sample (n < 30), and overlapped populations. At last, A total of 25 articles with 26 studies were selected for this meta-analysis.^[2,9,17–19,21–23,25–28,38–50]^ A flowchart of the study search and selection process is depicted in Figure 1.

### 3.2. Basic characteristics of eligible studies

The basic characteristics of the eligible studies are listed in Table 1. A total of 26 studies involving 2659 patients, with sample sizes ranging from 44 to 210. These studies were published between 2006 and 2019 and were conducted in 4 countries, including China, Japan, Germany, and Finland. The included studies referred to 13 types of solid cancers, including HCC, bladder cancer, lung cancer, osteosarcoma, colorectal cancer, gastric cancer, esophageal cancer, renal cell cancer, glioma, thyroid cancer, oral tong cancer, laryngeal cancer, and endometrial carcinoma. In 15 studies, securin expression was detected by IHC and 3 studies did it using PCR. A total of 17 articles with studies reported data about the association between securin expression and OS, whereas the correlation of securin expression and LNM was investigated in 13 studies. HRs and their 95% CIs were directly provided in 8 studies with 1 obtained by COX univariate analysis and 7 by multivariate analysis, while those in the other studies were calculated based on Kaplan–Meier curves. The Newcastle Ottawa Scale scores for all included studies were no <6, suggesting that their methodological quality was medium or high.

**Table 1:** Basic characteristics of the included studies.

Study	Country	Cancer type	Securin expression		Detection method	Definition of high PTTG1 expression	Tumor stage (case number)	Outcomes	NOS score
			High	Low					
Chen et al 2019	China	Esophageal cancer	41	32	IHC	IHC score ≥ 2	TNM stage I–II: 40 TNM stage III–IV: 33	LNM	6
Fujii et al 2006	Japan	Hepatocellular carcinoma	31	31	PCR	>Median value of the ratio of PTTG1 level in tumor to that in adjacent normal tissues	TNM stage I–II: 39 TNM stage III–IV: 23	OS^M^	6
Feng et al 2012	China	Endometrial carcinoma	79	45	IHC	IHC score > 0	TNM stage I–II: 101 TNM stage III–IV: 23	LNM	7
Feng et al 2016	China	Gastric cancer	105	99	IHC	IHC score ≥ 3	TNM stage I–II: 132 TNM stage III–IV: 65	LNM	7
GENKAI et al 2006	Japan	Glioma	22	22	IHC	IHC score > 1	Early stage: 52 Late stage: 41	OS^S^	6
Heikkinen et al 2016	Finland	Oral tongue cancer	27	66	IHC	Proportion of cancer cells stained positively ≥ 30%	NR	OS^S^	6
Ito et al 2008	Japan	Esophageal cancer	68	45	IHC	Staining score = 0 or 1	TNM stage I–II: 61 TNM stage III–IV: 52	OS^M^, LNM	7
Li et al 2013	China	Nonsmall cell lung cancer	77	69	IHC	Staining index score = 6 or 9	TNM stage I–II: 88 TNM stage III–IV: 58	OS^S^	6
Li et al 2015	China	Lung adenocarcinoma	27	23	IHC	NR	TNM stage I–II: 17 TNM stage III–IV: 33	LNM	6
Ma et al 2018	China	Laryngeal cancer	112	89	IHC	Staining index score ≥ 6	TNM stage I–II: 95 TNM stage III–IV: 115	OS^S^, LNM	6
Rehfeld et al 2006-1	Germany	Small cell lung cancer	27	109	IHC	Proportion of cancer cells stained positively > 0%	TNM stage I–II: 8 TNM stage III–IV: 125	OS^S^	6
Rehfeld et al 2006-2	Germany	Nonsmall cell lung cancer	37	54	IHC	Proportion of cancer cells stained positively > 0%	TNM stage I–II: 7 TNM stage III–IV: 84	OS^S^	6
Ren et al 2017	China	Colorectal cancer	67	51	IHC	IHC score > 4	TNM stage I–II: 38 TNM stage III–IV: 80	OS^M^	7
Sa´ez et al 2006	Spain	Thyroid cancer	35	13	IHC	Proportion of cancer cells stained positively > 25%	TNM stage I–II: 42; TNM stage III–IV: 18	LNM	6
Shibata et al 2006	Japan	Esophageal cancer	20	28	PCR	PTTG1 level relative to GAPDH > 0.113	TNM stage 0–III: 2 TNM stage IV: 16	OS^U^	6
Su et al 2006	China	Hepatocellular carcinoma	80	67	PCR	NR	TNM stage I–II: 62 TNM stage III–IV: 85	OS^S^	6
Wang et al 2016	China	Nonsmall cell lung cancer	71	65	IHC	IHC score > 4	TNM stage I–II: 56 TNM stage III–IV: 80	OS^M^	7
Wei et al 2015	China	Renal cell cancer	113	79	IHC	IHC score > 4	NR	OS^S^_,_ LNM	6
Wen et al 2015	China	Gastric cancer	54	26	IHC	IHC score ≥ 2	TNM stage I–II: 27 TNM stage III–IV: 53	LMN	6
Wu et al 2016	China	Osteosarcoma	55	16	IHC	IHC score ≥ 4	TNM stage I–II: 46 TNM stage III: 25	OS^S^	6
Xiang et al 2016	China	Bladder cancer	36	9	PCR	NR	TNM stage I–II: 23 TNM stage III: 23	LNM	6
Xu et al 2016	China	Gastric cancer	70	28	IHC	H-score	NR	OS^M^	7
Zhao et al 2009	China	Colorectal cancer	47	18	IHC	IHC score ≥ 3	Duke stage A–B: 40 Duke stage C–D: 25	LNM	6
Zeng et al 2017	China	Osteosarcoma	61	15	IHC	IHC score ≥ 4	TNM stage I–IIA: 31 TNM stage IIB–III: 45	OS^M^	7
Zhang et al 2014	China	Esophageal cancer	41	67	IHC	IHC score ≥ 4	TNM stage I–II: 66 TNM stage III–IV: 42	OS^M^_,_ LNM	7
Zhang et 2018	China	Esophageal cancer	52	24	IHC	Proportion of cancer cells stained positively > 0%	TNM stage I–II: 47 TNM stage III: 29	OS^S^_,_ LNM	6

IHC = immunohistochemical analysis, LNM = lymph node metastasis, M = multivariate analysis, NOS = Newcastle Ottawa Scale, OS = overall survival, PCR = polymerase chain reaction, S = survival curve, U = univariate analysis.

### 3.3. Combined analysis

**Figure 2.**
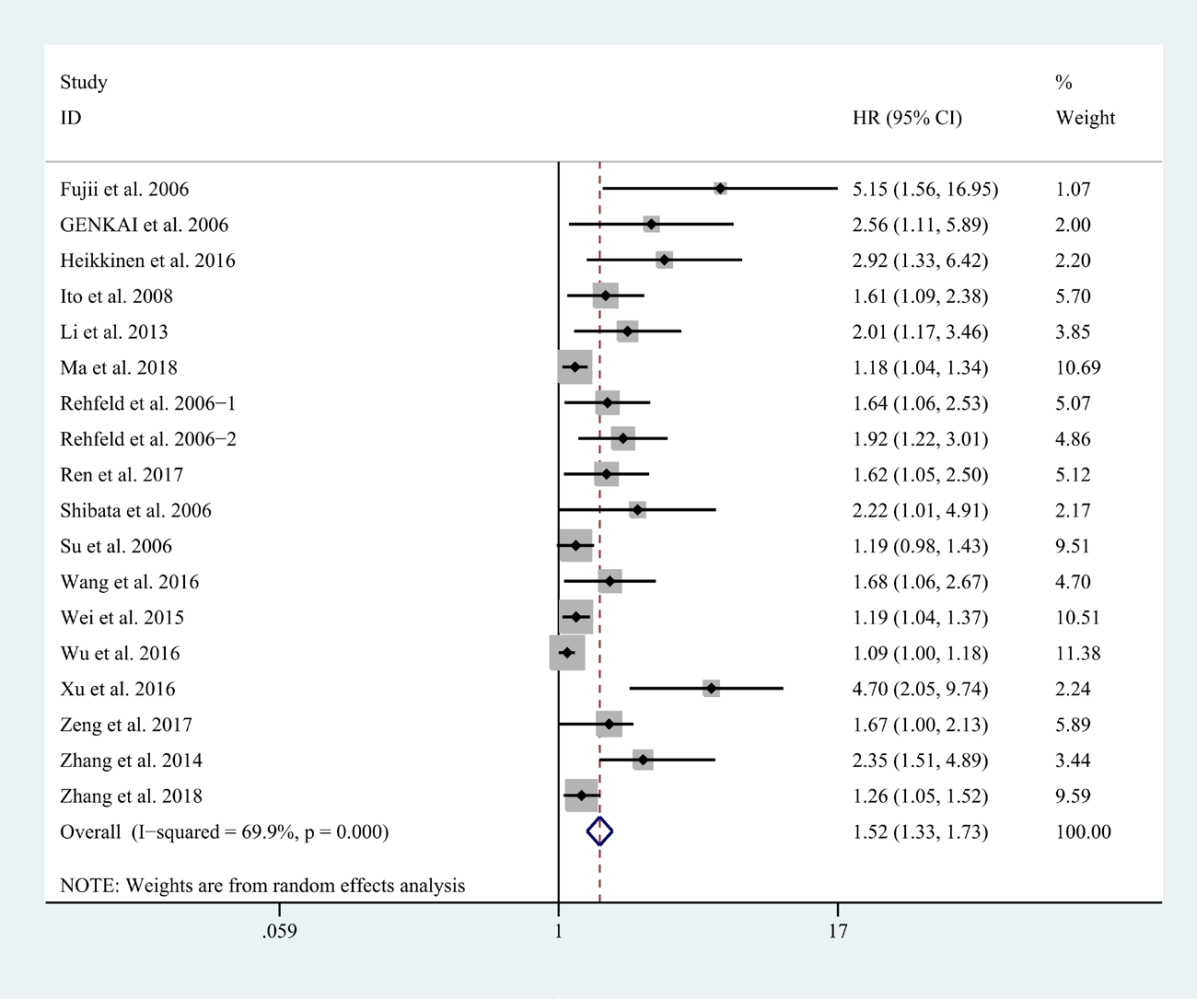
Forest plots of the association between securin expression and OS. CI = confidence interval, HR = hazard ratio, OS = overall survival.

HRs and 95% CIs for OS were directly or indirectly reported in 18 studies with 2569 patients. In view of the substantial heterogeneity (*I*^2 ^= 69.9%, *P* < .001), we used the random-effects model to synthesize the HRs and their CIs. The combined result indicated that high securin expression was closely correlated with poor OS (HR = 1.52, 95% CI: 1.33–1.73, *P* < .05, Fig. 2). With regard to LNM, 13 studies were included in the combined analysis. The fixed-effects model was used to calculate the OR and its CIs by combining data on LNM, as no extreme heterogeneity existed. The results showed a positive association between securin expression and LNM (OR = 2.96, 95% CI: 2.26–3.86, *P* < .05, Fig. 2).

Because significant heterogeneity was observed when data on OS were combined, we conducted subgroup analysis based on several variables, including country, cancer type, detection method, sample size, and analysis method, to investigate the sources of heterogeneity (Table 2). The results showed that substantial heterogeneity still existed in each subgroup of the detection methods (IHC and PCR), indicating that it might be closely correlated with heterogeneity. Notably, there was no statistically significant heterogeneity in the subgroups of Germany, lung cancer, esophageal cancer, sample size (n) > 109, and multivariate analysis, which suggests that country, cancer type, sample size, and analysis type might partly account for the substantial heterogeneity. Furthermore, in most subgroups, the conclusion that high securin expression predicted poor OS did not change. These results imply that our combined estimation of OS has good stability. Nevertheless, we found no significant correlation between securin expression and OS in the HCC, osteosarcoma, and PCR subgroups, which may be due to the small sample size.

**Table 2:** Association between securin expression and overall survival in subgroup analysis.

Variables	No. of studies	No. of patients	Combined HR	*P* value	Heterogeneity	
					*I*^2^ (%)	*P* value
**1. Country**						
China	11	1378	1.353 (1.193–1.535)	<.01	69.3	.023
Japan	4	267	2.118 (1.411–3.179)	<.01	24.7	.263
Germany	2	227	1.769 (1.293–2.42)	<.01	0	.622
**2. Cancer type**						
Hepatocellular carcinoma	2	209	2.189 (0.532–9.011)	.278	82.3	.017
Lung cancer	4	509	1.788 (1.415–2.26)	<.01	0	.92
Esophagus cancer	4	345	1.602 (1.187–2.162)	<.01	50.3	.11
Osteosarcoma	2	147	1.293 (0.859–1.944)	.218	78.3	.032
**3. Method**						
PCR	3	257	2.02 (0.921–4.429)	.079	73.9	.022
IHC	15	1708	1.519 (1.323–1.745)	<.01	71.2	.037
**4. Sample size**						
n ≤ 109	10	767	1.922 (1.46–2.529)	<.01	80.1	<.01
n > 109	8	1198	1.306 (1.168–1.459)	<.01	33.4	.162
**5. Analysis type**						
Multivariate	7	711	1.969 (1.522–2.547)	<.01	41.50	.114
Survival curve	10	1206	1.287 (1.15–1.441)	<.01	60.6	.015

HR = hazard ratios, IHC = immunohistochemistry, PCR = polymerase chain reaction.

### 3.4. Sensitivity analysis and publication bias

To evaluate the stability and reliability of our synthesized results, we conducted a sensitivity analysis by sequentially omitting each study. As illustrated in Figure 4A, no significant alterations were observed in the combined HR estimation for OS. Similarly, the combined OR of the LNM risk estimate did not significantly change without abrupt fluctuations (Fig. 4B). Publication bias was visually evaluated using a funnel plot and statistically assessed using the Egger’s test. With respect to OS, significant publication bias was detected using Egger’s test (*P* < .001). Accordingly, the funnel plot exhibited visual asymmetry (Fig. 5A). Therefore, we performed a trim-and-fill analysis to examine whether the bias significantly affected the reliability of our combined OS result. As shown in Figure 5B, 8 unpublished studies might be needed to neutralize the potential bias. After adjustment, the combined HR was slightly altered but remained statistically significant (HR = 1.3, 95% CI: 1.39–1.485). These results suggest that publication bias had a minimal effect on the combined OS result. For LNM, no substantial publication bias was found using Egger’s test (*P* = .091), which was verified by the symmetric shape of the funnel plot (Fig. 6). Taken together, sensitivity analysis and publication bias assessment indicated that the combined results were stable and reliable.

**Figure 3.**
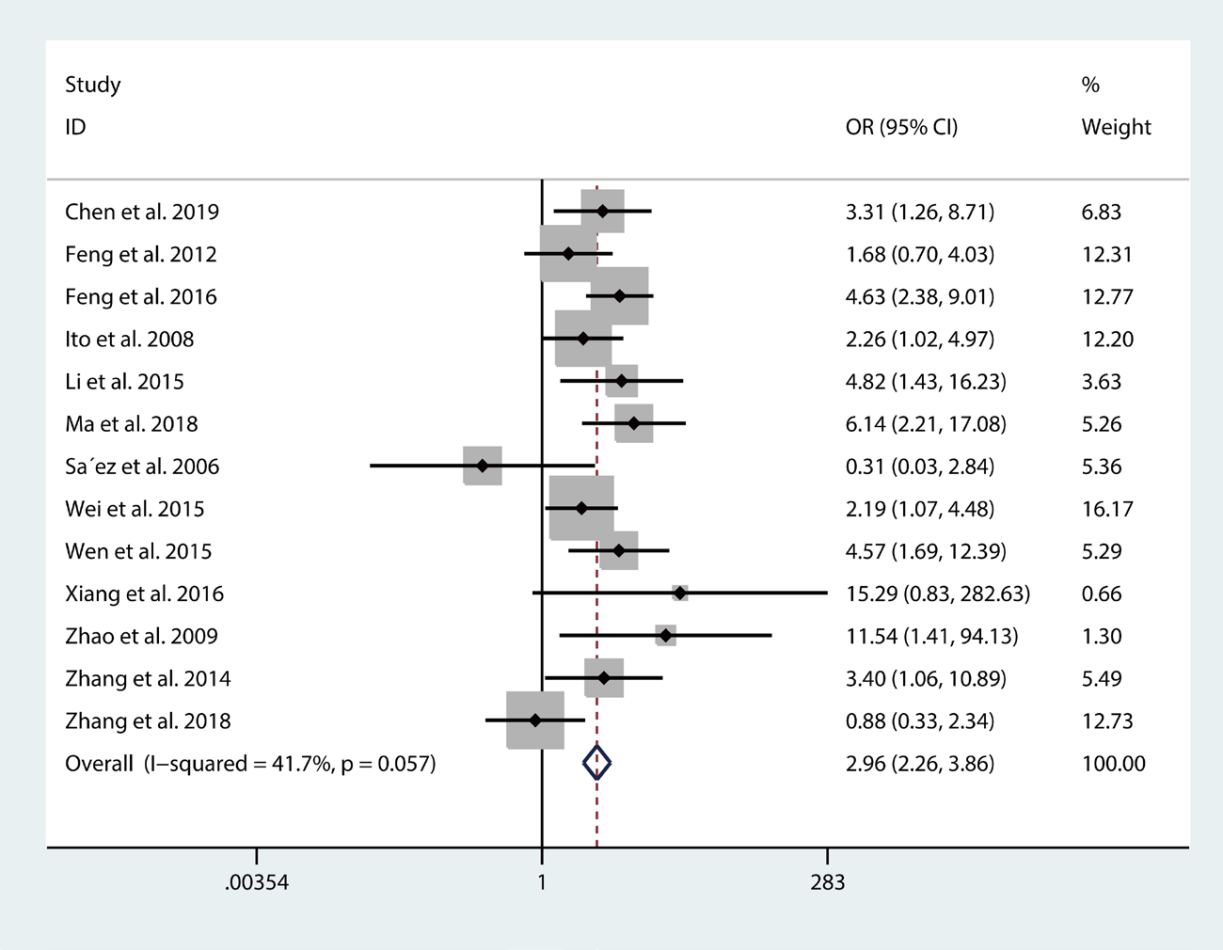
Forest plots of the association between high securin expression and LNM. CI = confidence interval, LNM = lymph node metastasis, OR = odd ratio.

### 3.5. Bioinformatics analysis based on TCGA dataset

We conducted GEPIA based on TCGA dataset to validate the results of our meta-analysis. In this bioinformatics analysis, 9500 cancer patients were included, and they were allocated into high and low groups according to the medium value of securin expression. GEPIA results indicated that high securin expression was significantly associated with poor OS (Fig. 7A) and disease-free survival (Fig. 7B). Overall, this bioinformatics analysis further confirmed the reliability of our meta-analysis.

**Figure 4.**
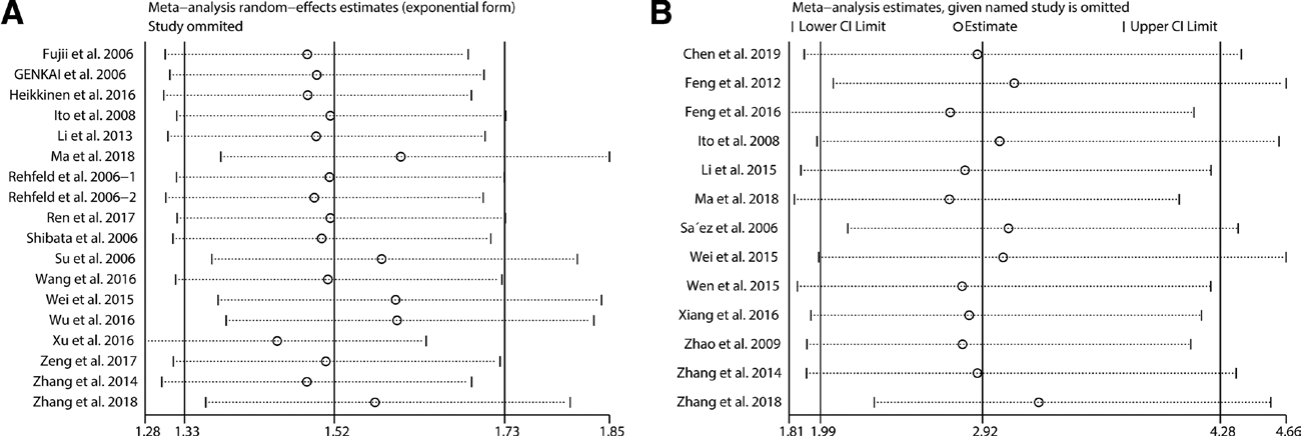
Sensitivity analysis of the association of high securin expression with OS (A) and LNM (B). LNM = lymph node metastasis, OS = overall survival.

**Figure 5.**
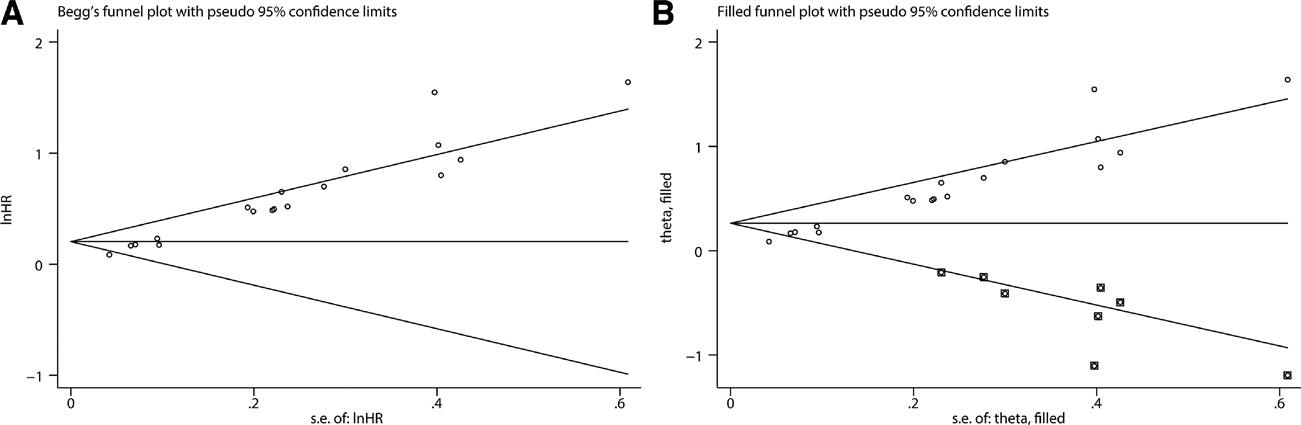
Begg’s funnel plot evaluating publication bias related to OS (A) and funnel plot adjusted with trim-and-fill methods for studies reporting OS (B). OS = overall survival.

**Figure 6.**
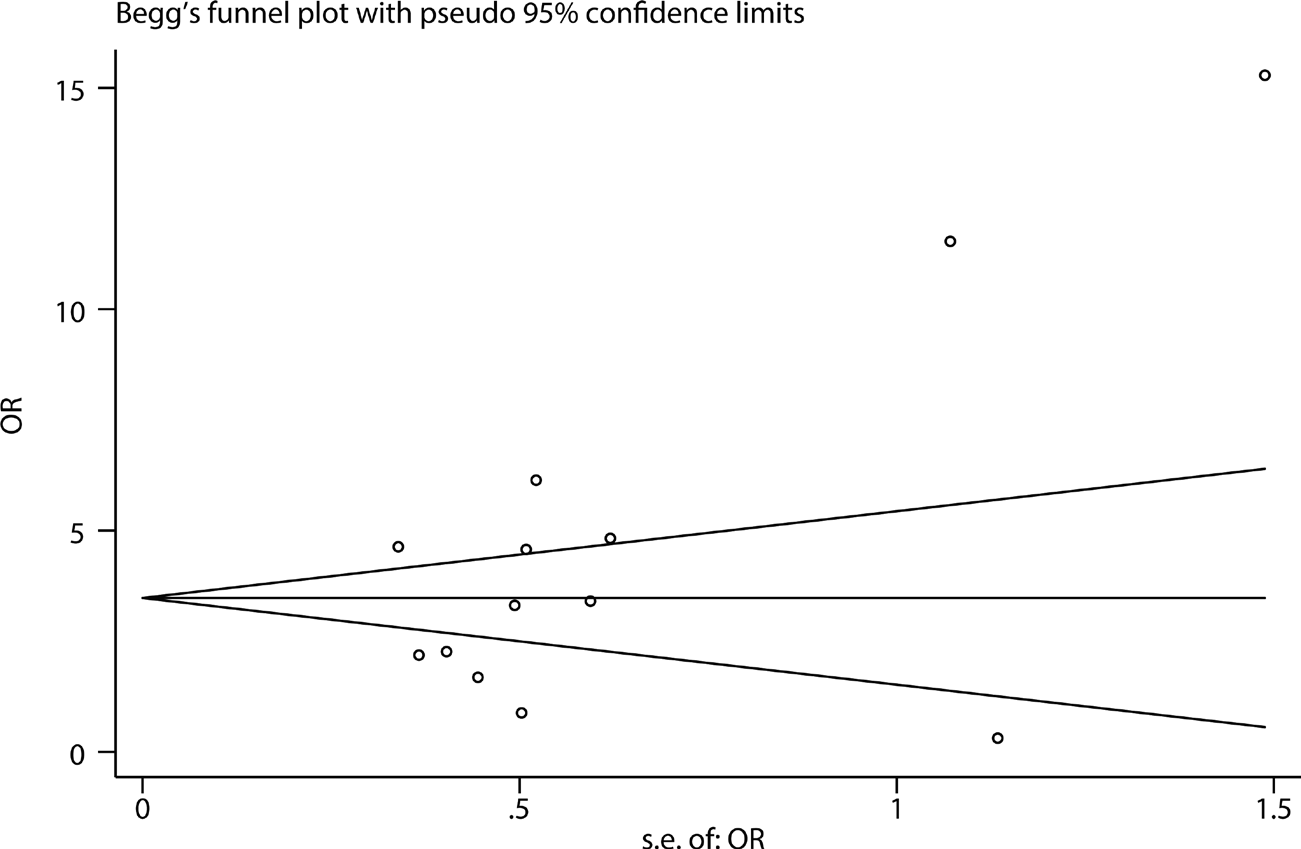
Begg’s funnel plot evaluating publication bias related to LNM. LNM = lymph node metastasis.

**Figure 7.**
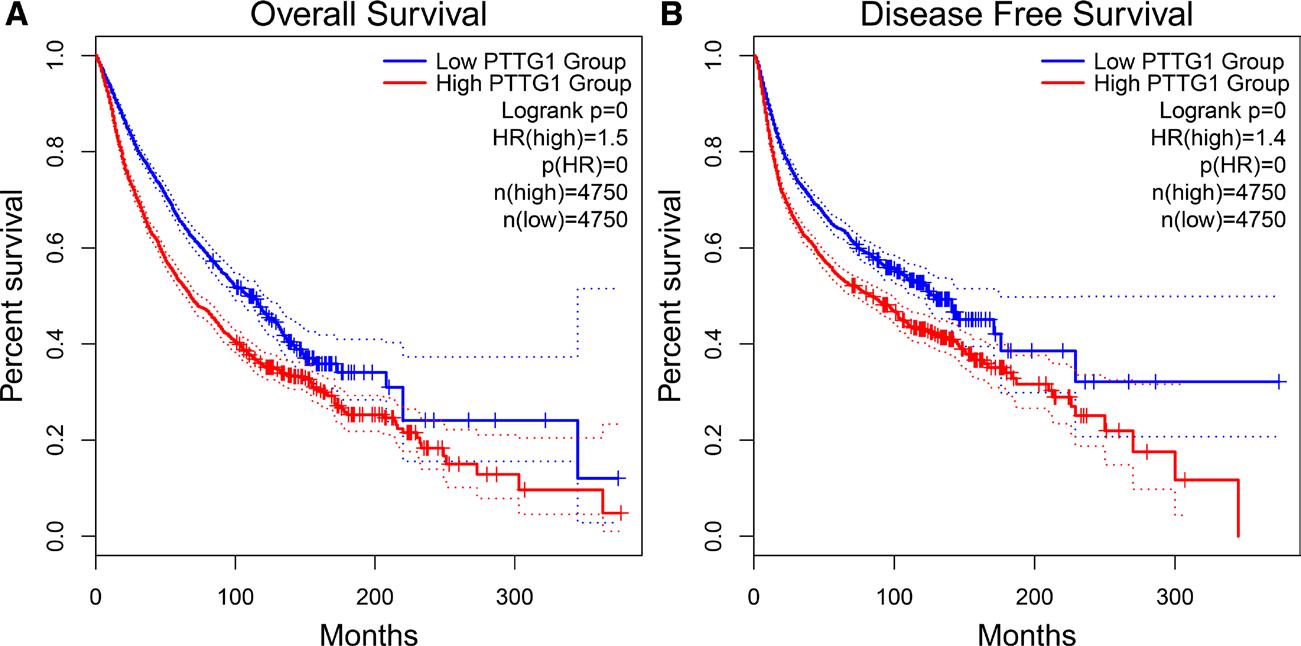
Survival curves generated from bioinformatics analysis for OS (A) and DFS (B). DFS = disease-free survival, OS = overall survival.

## 4. Discussion

The association between securin expression and survival outcomes of patients with solid cancers has been studied. Nevertheless, the results concerning the prognostic significance of securin was not consistent, which may be partly attributed to the small sample size in single-center set. Thus, we conducted this meta-analysis to comprehensively evaluate the prognostic value of securin expression in human cancers. It has been well established that tumor metastasis is an adverse event that severely affects the prognosis of patients with cancers.^[29,30]^ Given that, herein we also systematically assessed the association between securin expression and LNM through combining the data of the current literatures.

In this meta-analysis, we included 17 articles with 18 studies assessing the relationship between securin expression and OS in 2659 cancer patients. Additionally, 13 studies with 1377 patients were included to evaluate the potential of securin as a biomarker for LNM. Our combined results showed that high securin expression levels were significantly correlated with shorter OS. Meanwhile, we found a positive correlation between securin expression and LNM in solid cancers, which may partly account for the prognostic significance of securin expression, since tumor metastasis is a crucial risk factor for poor survival outcomes. Nevertheless, there was significant heterogeneity across the included studies when combining the data of OS. Many factors different among the included studies, such as country, cancer type, method of detecting securin expression, sample size, and analysis type of the prognostic value, may be responsible for the significant heterogeneity. To this end, we performed subgroup analyses based on these factors to explore the sources of heterogeneity. As a result, the statistically significant heterogeneity disappeared completely in subgroups by country, sample size, or analysis type, suggesting they may be the main sources of heterogeneity. Besides, no heterogeneity was not detected either in some of subgroups by cancer type or method, suggesting cancer type and method may also contribute to the heterogeneity in a degree. Notably, the close relationship between high securin expression and poor OS was observed in all subgroups, which indicated the robustness of our meta-analysis. At the same time, our sensitivity analyses also showed that the conclusions of the meta-analysis were not influenced by any single study, further verifying the stability of this meta-analysis. At last, publication bias assessment was performed to determine the reliability of this meta-analysis, and we found a significant publication bias in the meta-analysis of OS but not LNM. However, the trim-and-fill analysis indicated the publication bias exerted no obvious effects on the pooled result about OS. Overall, the results of subgroup analysis, sensitivity analysis, and publication bias supported the stability and reliability of our meta-analysis.

The multiple functions of securin in tumors may explain the positive association between its overexpression with the susceptibility to tumor metastasis and unfavorable prognosis (Table 3). In prostate cancer, upregulation of securin can promote tumor cell proliferation, growth, invasion, chemoresistance, and resistance to androgen-deprivation therapy, which may be partly attributed to the activation of the TGFβ1/SMAD3 pathway.^[51–56]^ In lung cancer, securin can activate focal adhesion kinase, AKT, and TGFβ1/SMAD3 pathways to facilitate proliferation, growth, survival, migration, invasion, EMT, chemoresistance, and radiation-induced immunosuppression.^[9,25,57–63]^ In breast cancer, securin induces cell cycle progression, proliferation, endocrine therapy resistance, EMT, and stemness by regulating p53, p27, and AKT signaling.^[64–70]^ In glioma, securin promotes tumor cell migration and invasion via upregulation of MMP-2 and MMP-9,^[71]^ and activates the AKT/mTOR pathway to induce tumor angiogenesis.^[72]^ Additionally, securin has been proposed to enhance glioma cell proliferation and inhibit apoptosis.^[73–75]^ In esophageal squamous cell cancer, securin upregulation contributed to cell motility and metastasis probably by increasing several members of the Ras and Rho families^[2]^ and via c-myc-mediated galectin-1 transactivation.^[11]^ Furthermore, securin was found to promote EMT of esophageal squamous cell cancer cells through binding to glioma-associated oncogene homolog1 promoter to activate its expression.^[12]^ In HCC, securin could negatively regulate tumor cell apoptosis via inactivation of p53 and p38 MAPK, and activation of AKT.^[13,14]^ Besides, securin was disclosed to promote HCC cell proliferation through upregulating c-myc.^[15]^Moreover, it has also been demonstrated that securin played a critical role in facilitating EMT, invasion, metastasis, and chemoresistance of HCC cells.^[76,77]^ In colorectal cancer, securin can lead to genetic instability by inhibiting Ku70 activity and disturbing the nonhomologous end-joining dsDNA repair pathway.^[16]^ In addition, accumulated evidence has shown that securin represses drug-induced senescence and apoptosis^[26,63,78,79]^ and contributes to the migration and invasion of colorectal cancer cells.^[79]^ In ovarian cancer, securin has been found to promote cell proliferation and growth by upregulating c-Myc and enhancing aerobic glycolysis.^[80]^ Securin can positively regulate ovarian cancer stem cell-associated self-renewal.^[3]^ In osteosarcoma, downregulation of securin inhibits the expression of p-Akt, MMP-2, and MMP-9 proteins, while increases the expression of p21 and E-cadherin proteins, thereby leading to cell cycle progression, proliferation, and invasion.^[81]^ In seminoma, securin facilitates cell migration and invasion by upregulating the expression of MMP-2 protein.^[82]^ In cholangiocarcinoma, silencing of securin repressed tumor cell proliferation induces cell cycle arrest and apoptosis by inactivating MAPK signaling pathway.^[10]^ In head and neck squamous cell carcinoma, deletion of securin can improve p53 protein stability in tumor cells, consequently suppressing cellular migration, invasion, and colony formation.^[83]^ Additionally, securin has been reported to participate in the progression of cervical cancer, oral squamous cell carcinoma, neuroblastoma, and bladder cancers by promoting EMT, migration, invasion, cell cycle progression, proliferation, and antiapoptosis of tumor cells.^[28,84–87]^ However, the molecular mechanisms underlying the role of securin in these cancer types remain largely unknown and should be studied in the future to develop securin-targeted drugs against these diseases.

**Table 3:** Roles of securin in different cancers.

Cancer type	Biological functions	Involved pathway	References
Prostate cancer	Promoting proliferation, growth, invasion, chemoresistance, resistance to androgen-deprivation therapy	TGFβ1/SMAD3	^[51–56]^
Lung cancer	Promoting proliferation, growth, survival, migration, invasion, EMT, chemoresistance, radiation-induced immunosuppression	FAK, AKT, TGFβ1/SMAD3	^[9,25,57–63]^
Breast cancer	Promoting cell cycle progression, proliferation, endocrine therapy resistance, EMT, stemness	p53, p27, AKT	^[64–70]^
Glioma	Promoting cell cycle progression, proliferation, growth, migration, invasion, EMT, angiogenesis; inhibiting apoptosis	MMP-2, MMP-9, AKT/mTOR	^[71–75]^
Esophageal cancer	Promoting migration, metastasis	Ras and Rho gene families, c-myc, GLI1	^[2,11,12]^
Hepatocellular carcinoma	Promoting proliferation, migration, invasion growth, metastasis, EMT, chemoresistance; inhibiting apoptosis	p53, AKT, p38, c-myc	^[13–15,76,77]^
Colorectal cancer	Promoting genetic instability, survival, growth, migration, invasion, metastasis; inhibiting apoptosis	Nonhomologous end-joining dsDNA repair pathway	^[16,26,63,78,79]^
Ovarian cancer	Promoting proliferation, aerobic glycolysis, growth, drug resistance, stemness, UV irradiation resistance	c-myc	^[3,80]^
Osteosarcoma	Promoting proliferation, cell cycle progression, invasion	AKT, MMP-2, MMP-9, p21, E-cadherin	^[81]^
Seminoma	Promoting migration, invasion	MMP-2	^[82]^
Cholangiocarcinoma	Promoting cell cycle progression, proliferation; inhibiting apoptosis	MAPK	^[10]^
Head and neck squamous cell carcinoma	Promoting proliferation, migration, invasion	p53	^[83]^
Bladder cancer	Promoting cell cycle progression, invasion, metastasis	Not explored	^[28]^
Neuroblastoma	Promoting cell cycle progression	Not explored	^[84]^
Oral squamous cell carcinoma	Promoting migration, invasion, EMT	Not explored	^[85]^
Cervical cancer	Promoting proliferation, growth, invasion; inhibiting apoptosis	Not reported	^[86,87]^

AKT = protein kinase B, EMT = epithelial-mesenchymal transition, FAK = focal adhesion kinase, GLI1 = glioma-associated oncogene homolog1, MAPK = mitogen activated protein kinase, MMP = matrix metalloproteinase.

There are some limitations in this meta-analysis and thereby the combined results should be interpreted with caution. First, the publication bias was substantial in our meta-analysis, probably exaggerating the overall pooled results. Usually, studies with negative results were less likely to be published versus those with positive results, which may partly account for the publication bias. Second, only articles written in English and Chinese were selected. Third, there was significant heterogeneity among the included studies, which may affect the stability and reliability of the combined results. Fourth, HRs in some studies were indirectly calculated using data extracted form survival curves, so a degree of errors may be inevitable. Fifth, owing to the insufficient data, we failed to carry out subgroup analyses to assess the prognostic significance of securin expression based on specific cancer types and differences that may arise from other covariates. Last but not least, the network analysis in the patients or Gene Set Enrichment Analysis cannot be performed to further evaluate the prognostic value of securin expression due to the unavailability of adequate original data, which may challenge the robustness of our evidence.

## 5. Conclusion

In summary, this meta-analysis suggests that high securin expression is closely correlated with unfavorable OS and positive LNM. Hence, securin may serve as a prognostic biomarker and therapeutic target for solid cancers. However, large-scale prospective homogeneous studies are needed to validate our results.

## Author contributions

**Conceptualization:** Dayang Zheng, Min Tang, Wangyan Zhou.

**Data curation:** Xiang Liu, Wei Zeng.

**Formal analysis:** Xiang Liu, Wei Zeng, Wangyan Zhou.

**Funding acquisition:** Wei Zeng.

**Investigation:** Wei Zeng.

**Methodology:** Xiang Liu, Wei Zeng.

**Software:** Xiang Liu, Wei Zeng.

**Supervision:** Dayang Zheng, Min Tang.

**Writing – original draft:** Xiang Liu, Wei Zeng, Wangyan Zhou.

**Writing – review & editing:** Dayang Zheng, Min Tang, Wangyan Zhou.

## Acknowledgments

We would like to thank Editage (www.editage.cn) for English language editing.
